# Subunit vaccines for *Acinetobacter baumannii*

**DOI:** 10.3389/fimmu.2022.1088130

**Published:** 2023-01-12

**Authors:** Ning Yang, Xiao Jin, Chenghua Zhu, Fenglin Gao, Zheqi Weng, Xingran Du, Ganzhu Feng

**Affiliations:** ^1^ Department of Respiratory and Critical Care Medicine, The Second Affiliated Hospital of Nanjing Medical University, Nanjing, Jiangsu, China; ^2^ The Second Clinical Medical School of Nanjing Medical University, Nanjing, Jiangsu, China; ^3^ Department of Infectious Disease, The Second Affiliated Hospital of Nanjing Medical University, Nanjing, Jiangsu, China

**Keywords:** *Acinetobacter baumannii*, subunit vaccine, immunity, adjuvant, immunization route

## Abstract

*Acinetobacter baumannii* is a gram-negative bacterium and a crucial opportunistic pathogen in hospitals. *A. baumannii* infection has become a challenging problem in clinical practice due to the increasing number of multidrug-resistant strains and their prevalence worldwide. Vaccines are effective tools to prevent and control *A. baumannii* infection. Many researchers are studying subunit vaccines against *A. baumannii*. Subunit vaccines have the advantages of high purity, safety, and stability, ease of production, and highly targeted induced immune responses. To date, no *A. baumannii* subunit vaccine candidate has entered clinical trials. This may be related to the easy degradation of subunit vaccines *in vivo* and weak immunogenicity. Using adjuvants or delivery vehicles to prepare subunit vaccines can slow down degradation and improve immunogenicity. The common immunization routes include intramuscular injection, subcutaneous injection, intraperitoneal injection and mucosal vaccination. The appropriate immunization method can also enhance the immune effect of subunit vaccines. Therefore, selecting an appropriate adjuvant and immunization method is essential for subunit vaccine research. This review summarizes the past exploration of *A. baumannii* subunit vaccines, hoping to guide current and future research on these vaccines.

## 1 Introduction

*Acinetobacter baumannii* is a strictly aerobic gram-negative bacillus, that exists widely in nature (1). *A. baumannii* is an opportunistic pathogen that can cause various nosocomial infections, including pneumonia, sepsis, meningitis, posttraumatic infections and urinary tract infections (2–4). This ESKAPE pathogen poses a significant threat to global public health because of its widespread drug resistance (5–8). In 2019, *A. baumannii* was responsible for more than 250 000 deaths associated with antimicrobial resistance (9). Effective antibiotic treatment is thus complicated and alternative therapeutic strategies are urgently needed, and vaccine is an effective tool to prevent and control *A. baumannii* infection (10–12).

Many researchers are currently developing subunit vaccines for *A. baumannii*. The subunit vaccine is a type of vaccine that contains active fragments of the pathogen to stimulate a protective immune response. Subunit vaccines have the advantages of high purity, safety, and stability, ease of production and highly targeted induced immune responses (13). Most of the *A. baumannii* candidate subunit vaccines are proteins, such as outer membrane protein A (OmpA). Polysaccharides, and outer membrane vesicles (OMVs) can also be used as subunit vaccines for *A. baumannii* (14). However, the subunit antigen fragment is small and lacks the tertiary structure of the protein. This fragment is easy to degrade *in vivo* and has weak immunogenicity. The use of adjuvants or delivery vehicles during subunit preparation can protect the antigen from degradation and enhance its immune efficacy (15, 16).

The development of reverse vaccinology, pan-genomics, core genomics, proteomics, immunoinformatics, and biophysical analyses have brought broad prospects in the exploration of potential antigen candidates of the subunit vaccine against *A. baumannii* (17–19). Afreenish Hassan et al. analyzed genetic data for all strains of *A. baumannii*, known as the pangenome, using proteomics and reverse vaccinology, thirteen types of proteins with high antigenicity were found in the conserved core genome (17). Among these four outer membrane proteins were prioritized: TonB-dependent siderphore receptor, OmpA family protein, type IV pilus biogenesis stability protein, and OprD family outer membrane porin (18). What’s more, recent vaccination trials used in silico computational approaches a type of epitope mapping of antigens to identify the prominent B-cell and T-cell epitopes (19, 20). To improve the immunogenicity of the epitope vaccine, it is important to combine multiple epitopes in series to form multi-epitope vaccines. Biophysical analysis was used to build a 3D model to test the stability of the vaccine and its ability to bind with host MHC-I, MHC-II, and toll-like receptors 4 (TLR-4) molecules on the surface of immune cells in the body (17, 18). Till now, epitope vaccines for *A. baumannii* candidate antigens, such as outer membrane protein 22, DcaP porin, MacB protein, and PcTPRs1 have been studied (20–23).

**Figure 1:**
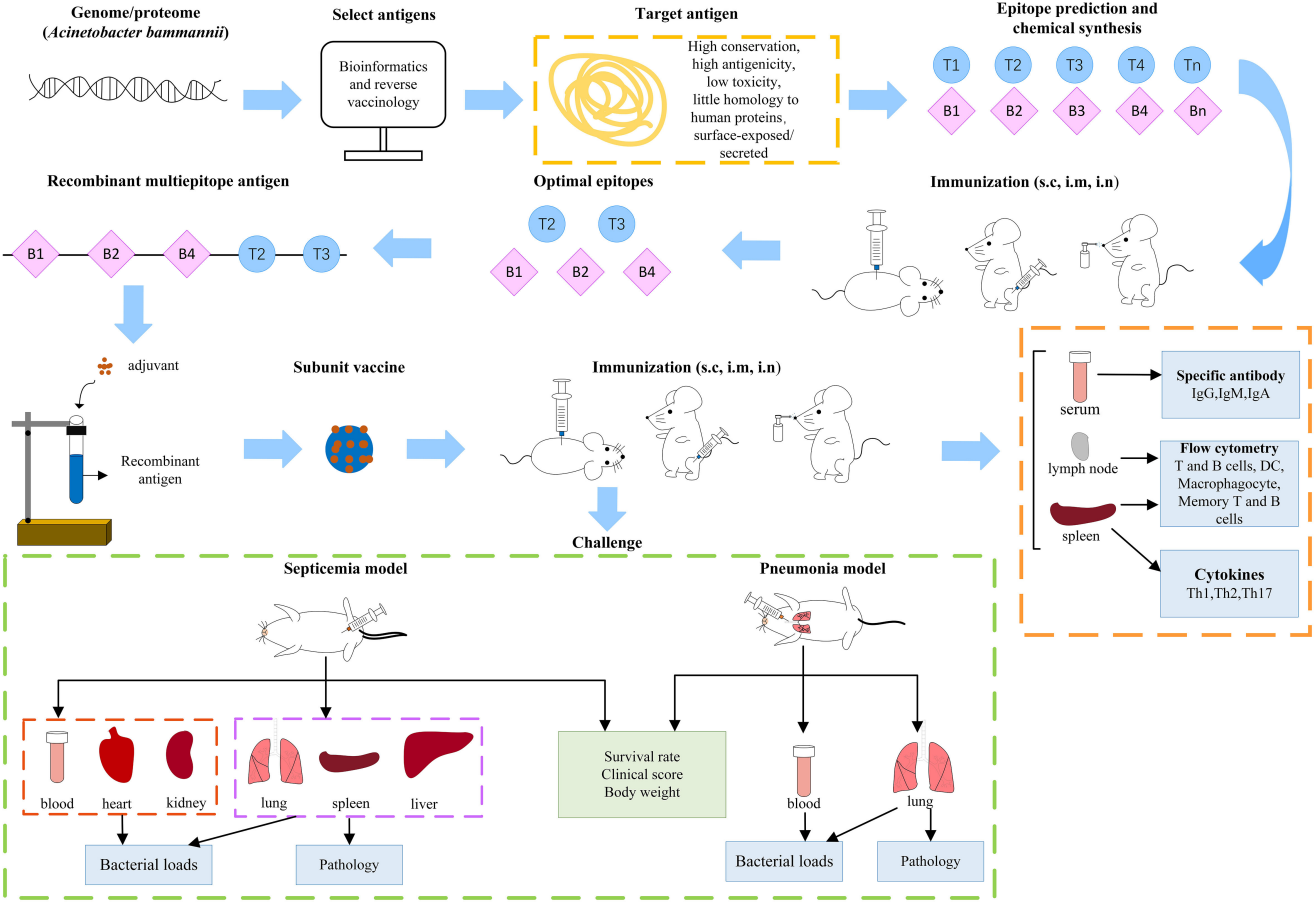
The general process of *Acinetobacter baumannii* subunit vaccine research. (s.c, subcutaneous; i.m, intramuscular; i.n, intranasal).

**Figure 2:**
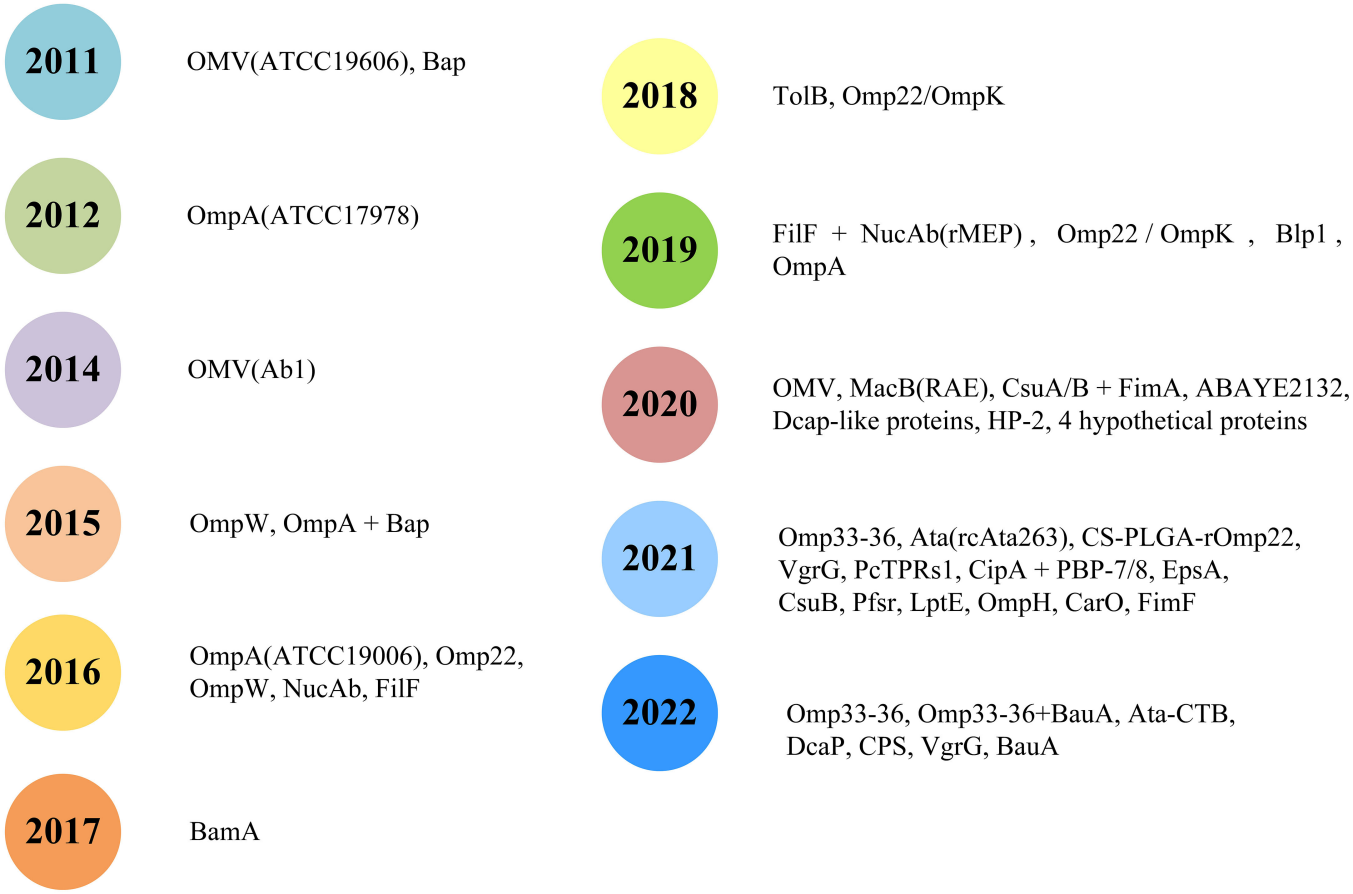
Subunit vaccines for *Acinetobacter baumanni*.

*A. baumannii* subunit vaccines (**Table 1**) that provide partial or complete protection have been previously studied. The development of new vaccines generally takes decades or even centuries (55). To date, no *A. baumannii* vaccine candidate has entered clinical trials. With the increase in multidrug-resistant strains and their prevalence worldwide, the development of new vaccines to prevent *A. baumannii* infection is urgently needed (5, 6). The general process for creating a subunit vaccine for *A. baumannii* is shown in **Figure 1**. This review summarizes past studies of *A. baumannii* subunit vaccines (**Figure 2**), hoping to provide guidance for current and future research on *A. baumannii* subunit vaccines.

**Table 1:** Candidate subunit vaccines for *Acinetobacter baumannii*.

		Immunization schedule					Challenge study					
Immunogen	Adjuvant	Mice	Day	Dose	Route	Model	Challenge strains	Day	Dose	Observation days after challenge	Survival	Reference
OmpA (ATCC17978)	Al(OH)3	BALB/c (>6m;6-10w)	0,21	3 μg	s.c	Sepsis (i.v)	HUMC1	35	2×10^7	28 days	50% 44.44%	(24)
OmpA (ATCC19006)	CT	BALB/c (6-8w)	0,21	10 μg	i.n	Sepsis (i.p)	Strain A Strain B Strain C Strain D Strain E	28	5×10^8 2×10^8 5×10^7 3×10^8 3×10^7	15 days	50% 40% 60% 100% 70%	(25)
OmpA+PKF OmpA PKF	Alhydrogel	C57BL/6 (6-8w)	0,14,28	25 μg	—	Sepsis (i.p)	ATCC19606	56	—	7 days	85.71% 66.6% 83.3%	(26)
Omp33-36	Freund’s adjuvant	BALB/c (6-8w)	0.14,28	20 μg	s.c	Sepsis (i.p) Pneumonia (i.n)	ATCC19606 AB022	42	4.8×10^4	4 days	42.85%-80% 57.14%-100%	(27)
Omp33-36 (ATCC19606) Omp33-36+BauA BauA	Freund’s adjuvant	BALB/c (4-6w)	1,14,28	20 μg	s.c	Sepsis (i.p)	ABI022	56	4.76×10^4	7 days	42.86% 66.67% 42.86%	(28)
Omp33-36	Freund’s adjuvant	BALB/c	0,14,28	20 μg	s.c	Sepsis (i.p)	ATCC19606	—	5×10^7 10×10^7	3 days	100% 0%	(29)
Trx-Omp22	Alum	ICR (6-8w)	0,14,28	50 μg 20 μg 10 μg	s.c	Sepsis (i.p)	Ab1	49	1×10^6	7 days	100% 33% 33%	(30)
CS-PLGA-rOmp22	CS, PLGA	BALB/c (6-8w)	0,14,28	40 μg	s.c	Pneumonia (i.t)	ATCC19606 CS-MDR-AB CRAB PDR-AB	42	2×10^8 1×10^9 5×10^8 5×10^8	7 days	83.33% 71.43% 66.67% 57.14%	(21)
Omp22/OmpK Omp22 OmpK	Freund’s adjuvant	BALB/c (6-8w)	1,14,21	20 μg	s.c	Sepsis (i.p)	ATCC19606	42	2×10^8	8 days	66.7% 37.5% 25%	(31)
Omp22/OmpK Omp22 OmpK	MF59	BALB/c (6-8w)	0,14,21	30 μg	i.t	Pneumonia (i.t)	ATCC19606	49	1×10^8	10 days	83.3% 41.7% 41.7%	(32)
Trx-OmpW	Alum	ICR (6-8w)	0,14,28	50 μg	s.c	Sepsis (i.p)	Ab1	49	1×10^6	7 days	100%	(33)
Ata (rcAta263)	Freund’s adjuvant	BALA/c	0,14,28,42	20 μg	s.c i.p i.n	Sepsis (i.p)	ATCC19606	56	1.2×10^6 1.2×10^6 4 × 10^8	7 days	66.667% 66.667% 100%	(34)
Ata-CTB	CTB	BALB/c (6w)	0,14,28	40 μg	s.c i.p s.c s.c	Sepsis (i.p)	ATCC17978 ATCC17978 ATCC17978 XH733	42	2×10^7 2×10^7 4.9×10^7 4.5×10^7	7 days	70% 100% 90% 60%	(35)
NucAb	Freund’s adjuvant	BALB/c (6-8w)	0,14,21	25 μg	i.p	Pneumonia (i.t)	ATCC19606	28	1×10^8	7 days	20%	(36)
BamA	Al(OH)3	BALB/c (6-8w)	1,14,28	20 μg	i.p	Pneumonia (i.n)	P-562	45	1×10^9	7 days	80%	(37)
Oma87 (BamA)	Freund’s adjuvant	BALB/c (6-8w)	0,14,28,42	20 μg	s.c	Sepsis (i.p)	ATCC19606	—	2×10^6 7×10^6	4 days	100% 25%	(38)
BauA Loop7 Loop875 Loop75 Loop85 Loop5 Loop8	Freund’s adjuvant	BALB/c (6-8w)	0,14,28	20 μg	s.c	Sepsis (i.p)	ATCC19606	43	1.8×10^6	7 days	100% 100% 83% 83% 67% 67% 50%	(39)
DcaP multiple-epitope vaccine of DcaP	Freund’s adjuvant	BALB/c (3-5w)	0,14,28	20 μg	s.c	Pneumonia (i.n)	ATCC19606	—	2×10^8.6	7 days	50% 33.3%	(22)
FilF	Freund’s adjuvant	BALB/c (6-8w)	0,14,21	20 μg	s.c	Pneumonia (i.t)	ATCC19606	29	1×10^8	7 days	50%	(40)
FilF + NucAb (rMEP)	Freund’s adjuvant	BALB/c (6-8w)	0,14,21	30 μg	s.c	Pneumonia (i.t)	ATCC19606	42	2×10^8	10 days	88.9%	(41)
PcTPRs1 subunit fragment of PcTPRs1	Freund’s adjuvant	BALB/c (4-5w)	0,14,28	20 μg	s.c	Pneumonia (i.n)	ATCC19606	—	2×10^8.6	7 days	50% 40%	(20)
ABAYE2132	Freund’s adjuvant	BALB/c (4-6w)	—	20 μg	s.c	Sepsis (i.p)	ATCC19606	—	8×10^8	3 days	100%	(42)
CsuA/B + FimA CsuA/B FimA	Freund’s adjuvant	BALB/c (6-8w)	0,14,28	10+10 μg 20 μg 20 μg	s.c	Sepsis (i.p)	ATCC19606	49	1.2×10^6	7 days	62.5% 37.5% 50%	(43)
Bap	Freund’s adjuvant	BALB/c (4-6w)	0,14,28	10 μg	—	Sepsis (i.p)	Kh0060	35	1×10^8 1×10^9 1×10^10 1×10^11 1×10^12 1×10^13	3 days	80% 100% 80% 80% 60% 60%	(44)
OmpA + Bap OmpA Bap	Al(OH)3	C57BL/6 (6-8w)	0,14,28	75 μg 50 μg 25 μg	s.c	Sepsis (i.p)	ATCC19606 MDR AB-44 ATCC19606 ATCC19606	42	1.14 × 10^5 7.5 × 10^3 1.14 × 10^5 1.14 × 10^5	7 days	100% 85.71% 71.43% 42.86%	(45)
Blp1	Freund’s adjuvant	BALB/c (8-12w)	0,14,28	2 μg	i.m	Sepsis (i.p)	AbIC I	42	1×10^8	7 days	60%	(46)
VgrG	Freund’s adjuvant	BALB/c (6-8w)	0,14,28	20 μg	s.c	Sepsis (i.p)	ATCC19606	56	1.2×10^6	7 days	33.3%	(47)
VgrG	Freund’s adjuvant	BALB/c (6-8w)	0,15,30	20 μg	s.c	Sepsis (i.p)	ATCC19606	—	1.9×10^8 2.4×10^8 3.2×10^8	3 days	100% 75% 0%	(48)
MacB (RAE)	Freund’s adjuvant	BALB/c (6w)	0,14,28,42	200 μg	—	Sepsis (i.p)	ATCC17978	56	1×10^7	14 days	0%	(23)
CipA + PBP-7/8 CipA PBP-7/8	MPLA	C57BL/6 (6w)	0,14,28	30 μg	s.c	Sepsis (i.p)	ATCC19606	42	2.12×10^5	7 days	80% 60% 60%	(49)
CPS	Freund’s adjuvant calcium phosphate	BALB/c (6-8w)	0,14,28	50 μg	s.c	Sepsis (i.p)	K9	42	1×10^8	10 days	100% 70%	(50)
C-CPS CPS	Al(OH)3	BALB/c (6w)	0,14,28	20 μg	s.c	Sepsis (i.p)	ATCC19606 MDR-ZJ06	42	4.9×10^7 1.2×10^7	7 days	100% 80% 30% 30%	(51)
OMV (ATCC19606)	Adjuphos	C57BL/6 (6-8w)	0,14	50 μg	i.m	Sepsis (i.p)	ATCC19606 Ab-154 113-16	21	4.5×10^5 1.7×10^6 2×10^6	7 days	100% 87.5% 100%	(52)
OMV (Ab1)	Alum	ICR (6-8w)	0,15,36	50 μg	i.m	Sepsis (i.p)	Ab1	47	5.5×10^5	7 days	73.3%	(53)
OMV	Alhydrogel	C57BL/6 (6-8w)	0,14	10 μg 100 μg	i.m	Sepsis (i.p)	ATCC19606	21	2×10^6	7 days	75% 100%	(54)

CT, cholera toxin; CTB,cholera toxin B subunit; CS, chitosan; PLGA, poly lactic-glycolic acid; MPLA, Monophosphoryl lipid A; s.c, subcutaneous; i.p, intraperitoneal; i.n, intranasal; i.v, intravenous; i.t, intratracheal; i.m, Intramuscular.

## 2 Candidate subunit vaccines for *Acinetobacter baumannii*

Many subunit vaccine antigens for *A. baumannii* have been explored with varying degree of success. Around twenty *A. baumannii* subunit vaccines have been evaluated *in vivo* for their immunogenicity and protective effects. Among them, the most studied antigens are outer membrane proteins (OmpA, Omp33-36, Omp22, OmpW, and Ata, et al.), fimbrial proteins (CsuA/B and FimA), and capsular polysaccharide (11, 14, 56). Outer membrane vesicles (OMVs) have been also studied in recent years. In addition, over ten novel antigens, such as Pfsr, LptE, OmpH, CarO and FimF, predicted by reverse vaccinology are worthy of further exploration (57, 58). The candidate antigens for *A. baumannii* subunit vaccines can be divided into three categories: proteins, polysaccharides, and outer membrane vesicles.

### 2.1 Proteins

#### 2.1.1 Outer membrane proteins

##### 2.1.1.1 OmpA

Outer membrane protein A (OmpA), also known as Omp38, with a molecular weight of 38 kDa, is one of the most abundant porins in the outer membrane of *A. baumannii* (59). OmpA plays a key role in regulating the adhesion, invasiveness, biofilm formation, apoptosis and the associated host immune response of *A. baumannii* (60–65). Additionally, OmpA regulates autophagy (66). In recent years, with improved understanding of the natural structure of OmpA, researchers have found that the amino acids of OmpA from various clinical isolates of *A. baumannii* are highly conserved (>99%), and they are of a different origin compared to the human proteome (24).

Some studies have shown that immunization with OmpA can protect against *A. baumannii* infection. Mice were passively immunized with a yolk antibody (IgY) specific to OmpA, which provided partial protection. IgY prolonged the survival of mice attacked by *A. baumannii* (67, 68). Mice were subcutaneously immunized with recombinant OmpA (rOmpA). After challenge with *A. baumannii*, the survival rate of actively immunized mice was more than 40%, and that of passively immunized mice was up to 90% (24). Increased doses of the rOmpA vaccine resulted in enhanced type 2 immune responses, increased IL-4-induced T-cell epitope spread and decreased levels of IFN-γ-induced epitopes (69). When mice were intranasally immunized with rOmpA, anti-OmpA IgG was produced in the serum, and anti-OmpA IgA was produced in saliva. After intraperitoneal immunization of mice with rOmpA and stimulation with different doses of *A. baumannii*, the 15-day survival rate of the mice was 40%-100% (25). The total IgG concentration in the serum of mice immunized with OmpA and the serine protease PKF was significantly increased. This protein can provide 85.71% protection against *A. baumannii* (26). The serum IgG antibody level was significantly increased in mice immunized with OmpA and BauA. After stimulation with *A. baumannii*, the bacterial loads in the lung, spleen and liver of immunized mice were significantly lower than those in the control group (70).

OmpA is an important protein in *A. baumannii*. This protein is highly conserved, exhibiting high immunogenicity and low endotoxin levels after treatment. OmpA can induce a robust protective effect against *A. baumannii* in mice. Therefore, OmpA is considered the most promising subunit vaccine candidate and the antigen with the most clinical translational value. However, OmpA has not yet entered clinical trials which may be related to its toxicity. Luo et al. used a detoxifying gel endotoxin removal column to greatly reduce OmpA endotoxin level, but 1-4 EU/3μg of endotoxin remained (24). The measurement of endotoxin level has been limited to animal studies. Immune cell membranes coated or fused with OmpA may neutralize OmpA toxicity and improve OmpA immunogenicity.

##### 2.1.1.2 Omp33-36

Outer membrane protein 33-36, known as Omp33 or Omp34 in some *Acinetobacter* species, is another outer membrane virulence factor involved in host cell adhesion. Omp33-36, OmpA and TonB are collectively known as fibronectin-binding proteins (FBPs) (71). Omp33-36 is associated with the adhesion and invasion abilities, cytotoxicity and metabolic adaptability of *A. baumannii* (72). Omp33-36 can promote apoptosis and regulate autophagy in human cells (73, 74).

Omp33-36 is recognized explicitly by IgM, IgA, and IgG from patients infected with *A. baumannii*. Omp33-36 did not cross-react with sera from patients infected with pathogens other than *A. baumannii* (75). Omp33-36 sequences showed ≥98% identity among more than 1670 strains of *A. baumannii* (76). Passive immunization with a yolk antibody (IgY) specific to Omp33-36 successfully protected a mouse model from pneumonia caused by *A. baumannii* (67, 68, 77). The conserved exposure loop 3 of Omp33-36 combined with the loopless-C-lobe (LCL) of TbpB of *Neisseria meningitidis* was used to actively immunize mice. Mice were stimulated with lethal doses of *A. baumannii*. In the sepsis model, the 96-hour survival rate of mice was 42.85%-80%. In the pneumonia model, the survival rate of mice was 57.14%-100% (27). The immunogenic loops of Omp33-36 are nontoxic. Mice were immunized with a hybrid antigen composed of BauA and immunogenic loops of Omp33-36 and LCL. The 7-day survival rate of mice vaccinated with hybrid antigens (71.43%) was higher than that of mice immunized with Omp33-36 immunogenic loop alone (42.86%) (28). The survival rate of mice immunized with recombinant Omp33-36 (rOmp33-36) was 100% under stimulation with *A. baumannii* (29).

Omp33-36 is highly conserved, nontoxic, and has high immunogenicity in *A. baumannii*, providing partial or complete protection against *A. baumannii* infection. Omp33-36 has the potential for clinical transformation. This protein is under preclinical study and may be related to its immunogenicity. Omp33-36 does not provide complete protection in all kinds of challenge models. Novel adjuvants, such as nanomaterials and bacterial outer membrane vesicles, may improve the immunogenicity of Omp33-36.

##### 2.1.1.3 Omp22

Outer membrane protein 22 (Omp22) is 217 amino acids in length and exhibits >95% conservation among 851 A*. baumannii* strains, showing little homology to human proteins (30). Omp22 is slightly toxic to mammalian cells, and high doses of Omp22 do not cause obvious pathological changes in mice. Mice were immunized subcutaneously with a high dose of recombinant Trx-Omp22 fusion protein. Survival reached 100% at 7 days after challenge with lethal doses of *A. baumannii*, whereas low doses showed protection in one-third of the mice (30). Recombinant Omp22 (rOmp22) was encapsulated with chitosan (CS) and polylactic-co-glycolic acid (PLGA) to form the CS-PLGA-rOmp22 nanovaccine. The 7-day survival rate of mice immunized subcutaneously with CS-PLGA-rOmp22 was 57.14%-83.3% in the pneumonia model (21). The fusion protein Omp22/OmpK was formed by using Omp22 and OmpK. Mice immunized subcutaneously with Omp22/OmpK and Freund’s adjuvant had a survival rate of 66.7% under *A. baumannii* attack (31). The combination of Omp22/OmpK and MF59 was injected into the trachea of mice, and the protective effect was found to be stronger than that of OmpK alone. The survival rate was 83.3% at 10 days after stimulation (32). Omp22 is highly conserved with low toxicity and some immunogenicity. Omp22 is not highly protective in mice, but its protective effect can be improved by changing the type of adjuvant. Omp22 is a valuable candidate for further study.

##### 2.1.1.4 OmpW

Outer membrane protein W (OmpW) is an eight-stranded β-barrel pore protein that forms a channel through the outer membrane to absorb small hydrophobic molecules (78). Hypoxia leads to downregulation of OmpW, resulting in decreased adhesion and invasion of *A. baumannii* in human lung epithelial cells and reduced biofilm formation (79). OmpW is also involved in colistin binding and plays an important role in regulating bacterial iron homeostasis (33, 80). The homology of OmpW among 804 reported *A. baumannii* strains was > 91%. OmpW had a slight inhibitory effect on 293FT and A549 cells, indicating that OmpW may affect the growth of normal cells and tumor cells. In a sepsis model, both active and passive immunization with recombinant Trx-OmpW protein were remarkably effective against *A. baumannii* infection. Seven days after *A. baumannii* challenge, the survival rate of actively immunized mice was 100% and that of passively immunized mice was 83.3% (33). OmpW is conserved among *A. baumannii* strains and has high immunogenicity, with the ability to protect against *A. baumannii* infection. OmpW is one of the candidate subunit vaccines of *A. baumannii*. However, further research is needed to eliminate or attenuate the virulence of OmpW, for example, by selecting some reagents that can neutralize or remove the toxin. Additional research is needed before the use of OmpW can be translated to clinical trials.

##### 2.1.1.5 Ata

*Acinetobacter* trimeric autotransporter adhesin (Ata) belongs to the superfamily of trimeric autotransporter adhesins, which are crucial virulence factors in *A. baumannii*. Ata mediates adhesion and invasion, induces apoptosis and contributes to pathogenicity *in vivo* (81, 82). Ata also participates in biofilm formation and binds to various extracellular matrix/basement membrane (ECM/BM) components (82).

Ata is a potential vaccine target. The antibodies against Ata have a strong opsonization effect on *A. baumannii*, with low to moderate killing activity against four *A. baumannii* strains (83). A conserved 263-amino-acid fragment from the C-terminus of Ata could elicit specific antibody responses and protect against challenges in mice. Mice were immunized with Ata fragments by abdominal, subcutaneous, and nasal mucosal immunization. When challenged with *A. baumannii*, the survival rate of mice immunized in the abdominal and subcutaneous areas was 66%, and that of mice immunized in the nasal mucosa was 100% (34). A short peptide containing only 39 amino acids located in the extracellular region of the C-terminal region of Ata was fused with the B subunit of cholera toxin (CTB), and experiments showed that this protein had no systemic toxicity. Subcutaneous and intraperitoneal immunization of mice with the fusion protein elicited T1 and T2 immune responses *in vivo*. When challenged with *A. baumannii*, the survival rate of mice immunized subcutaneously was 60% to 90% and that of mice immunized intraperitoneally was 100% (35).

Ata has high immunogenicity, no toxicity, and good safety and can prevent *A. baumannii* infection. Ata is one of the candidate subunit vaccines for *A. baumannii* and has value in clinical translation. Its effect on nasal mucosal immunity is promising. Mice immunized with Ata *via* nasal mucosa had a strong immune effect and strong protective effect on mice. The long-term protective effects of Ata should be examined in future studies. Research on the protective effect of nasally immunized mice against various types of *A. baumannii* strains will be of great significance.

##### 2.1.1.6 NucAb

Outer membrane nuclease (NucAb) is a protein in the outer membrane of *A. baumannii*. As the name suggests, this protein has nuclease activity. Both gram-negative and gram-positive bacteria can produce outer membrane nucleases, which may be related to bacterial virulence and survival in harsh environments (84–89). NucAb has all the characteristics of a vaccine candidate, such as outer membrane localization, and lack of homology to human proteins. The NucAb gene was found to be highly conserved (100%) among 40 clinical isolates of *A. baumannii* and showed more than 98% conservation among sequenced *Acinetobacter* strains present in the NCBI database (36). The endotoxin level of recombinant NucAb was low (< 1 EU/ml). Mice immunized with recombinant NucAb showed significant inhibition of inflammation in lung histopathological examination. Lung and serum levels of proinflammatory cytokines (TNF-α and IL-6) were significantly reduced, and those of anti-inflammatory (IL-10) cytokines were increased. NucAb is a candidate subunit vaccine against *A. baumannii*. NucAb is highly conserved, with good safety and some immunogenicity. However, the survival rate was only 20% in the group of actively immunized mice, and the survival rate of passively immunized mice was approximately 40% after *A. baumannii* infection (36). Therefore, we think NucAb has a low probability of being clinically translatable.

##### 2.1.1.7 BamA

BamA is an outer membrane β-barrel assembly protein responsible for organizing protein complex on the outer membrane of bacteria (37, 90, 91). The formation and assembly of outer membrane proteins on bacterial membranes are related to the action of the Bam protein complex (91). Among these proteins, BamA represents a potential target. BamA is anchored to the cell membrane and has a small extracellular portion that can generate immunogenic epitopes (92). The sequence identity of BamA in *A. baumannii* strains was 92.3% to 99.9%. Mice immunized with recombinant BamA produced IgG with high titers. In a mouse pneumonia model, the 7-day survival rate was 80% in actively immunized mice and 60% in passively immunized mice. Compared with unimmunized mice, immunized mice had a lower bacterial load in their lungs and significantly reduced the levels of the serum proinflammatory factors IL-6 and IL-1β (37). In silico analyses revealed that Oma87 is the same as BamA. Mice immunized with recombinant Oma87 (rOma87) showed a high specific IgG titer in serum. Immunized mice stimulated with twice the lethal dose of *A. baumannii* had a survival rate of 100%. Mice immunized with seven times the lethal dose of *A. baumannii* had a survival rate of 25% (38). As a candidate subunit vaccine, BamA has high conservation and immunogenicity and low toxicity. BamA can provide vital protection to immunized mice, with potential for clinical translation. The immunogenicity of BamA has room for improvement. The T-cell and B-cell epitopes of BamA were screened by bioinformatics, and the recombinant protein was constructed to immunize mice, which may improve the immunogenicity of BamA.

##### 2.1.1.8 BauA

Baumannii acinetobactin utilization (BauA) is the most important member of the iron-regulated outer membrane proteins (IROMPs) family of *A. baumannii*. BauA plays a key role in the absorption of acinetobactin and iron complexes under iron-restricted conditions (93, 94). This protein is a monomer, composed of the cork and the β-barrel domains. The barrel is composed of 22 antiparallel transmembrane β-chains. The N-terminal domain, called the cork occludes the β-barrel (95). BauA is conserved among members of *Acinetobacter* genus (96). The antibody titers in the serum of mice immunized with recombinant BauA (rBauA) were significantly higher than those in the control group (97, 98). Exposure loop 7 of BauA was combined with LCL to form a hybrid antigen. Immunizing mice with hybrid antigens resulted in rather high antigen titers. BauA can provide complete protection against *A. baumannii* and is a promising candidate subunit vaccine (39).

##### 2.1.1.9 DcaP

DcaP is a porin of *A. baumannii* that is involved in biofilm formation (99). This protein is the most abundant dispersal channel for *A. baumannii* during rodent infection. The X-ray crystal structure of DcaP showed a trimeric pore structure. DcaP may be involved in the uptake of clinically relevant negatively charged β-lactamase inhibitors, such as sulbactam and tazobactam (100). The DcaP sequence was more than 90% identical among the 1450 A*. baumannii* strains studied. There was no similarity between DcaP and human or mouse proteins. The survival rates of active and passive DcaP-immunized mice infected with two times the lethal dose of *A. baumannii* strains were 50% and 66.7%, respectively. Mice actively and passively immunized with the multiepitope vaccine of DcaP showed 33.3% and 50% survival after challenge with twice the lethal dose of *A. baumannii* (22). DcaP, a candidate subunit vaccine, is highly conserved and has a different origin from human proteins. This protein exhibits some immunogenicity and is not a toxin or an allergen. DcaP did not provide sufficient protection to immunized mice, and the survival rate of immunized mice stimulated by *A. baumannii* was not high. The prospects for clinical translation of DcaP are low.

##### 2.1.1.10 FilF

FilF is a putative pilus assembly protein located in the outer membrane of *A. baumannii*. It is highly conserved among *A. baumannii* strains, but its function remains unclear (40). This protein contains 641 amino acids, 20 of which form signal peptides for localization to the outer membrane. The level of FilF endotoxin was less than 1 EU/ml. Immunization of mice with FilF by subcutaneous injection induced specific IgG production and significantly decreased the levels of the proinflammatory factors TNF-α, IL-6, IL-33, IFN-γ, and IL-1β. When treated with lethal doses of *A. baumannii*, the bacterial load in the lung tissue of mice was significantly reduced compared with that in the control group, and the survival rate of mice was up to 50%, indicating that FilF provided immunological protection against *A. baumannii* infection (40). The T-cell and B-cell epitopes of FilF and NucAb were screened by bioinformatics software to generate the recombinant multiepitope assembly peptide (rMEP). Immunizing mice with rMEP could induce high levels of IgG antibodies and provide effective protection (88.9%) against a lethal dose of *A. baumannii* (41). FilF is conserved, immunogenic, and weakly toxic. FilF has a weak protective effect on mice, and the survival rate of mice under *A. baumannii* attack is not very high. Therefore, the possibility of clinical translation is low.

##### 2.1.1.11 PcTPRs1

PcTPRs1 is a protein containing a tetrapeptide repeat (TPR) that is associated with bacterial pathogenicity and virulence (20, 101, 102). PcTPRs1 is also involved in a variety of biological processes, such as gene regulation, cell cycle regulation, transfer of bacterial virulence factors to host cells, binding to host cells, and inhibition of phagolysosome maturation (103–105). PcTPRs1 is an outer membrane protein that is very highly conserved (identity>90%) in *A. baumannii*. Mice immunized with PcTPRs1 and its subunits had survival rates of 50% and 40%, respectively, after stimulation with twice the lethal dose of *A. baumannii*. Immunized mice produce specific IgG antibodies. Mice passively immunized with PcTPRs1 and its subunits had survival rates of 66.7% and 50%, respectively (20). PcTPRs1, a candidate subunit vaccine of *A. baumannii*, is very highly conserved and is immunogenic. PcTPRs1 did not have a strong protective effect on mice. Therefore, the probability of clinical translation is low.

#### 2.1.2 Fimbrial proteins

##### 2.1.2.1 ABAYE2132

ABAYE2132 is a fimbrial protein associated with the adhesion, invasion, biofilm formation and motility of *A. baumannii.* When ABAYE2132-resistant serum was cultured with *A. baumannii* and A549 cells, the adhesion of *A. baumannii* to A549 cells was reduced by 40% (42). ABAYE2132 is highly conserved, with an identity of more than 99% (42, 106). The full-length protein sequence consists of 209 amino acids. Immunized of mice with ABAYE2132 provided complete protection against *A. baumannii* stimulation, and the bacterial loads in the lungs, liver and spleen of the immunized mice were significantly lower than those in the control group (42).

##### 2.1.2.2 CsuA/B and FimA

Chaperone-Usher (CU) pili is a virulence factor involved in bacterial adhesion; CsuA/B and FimA are important components of CU pili (107–110). CsuA/B and FimA are highly conserved among *A. baumannii* strains. The identity of CsuA/B in 2927 A*. baumannii* strains was ≥99.44%, and the query coverage (QC) was ≥98%. The QC of FimA among 2300 strains of *A. baumannii* was 100% (43). The two antigens, CsuA/B and FimA, are safe and do not cause toxicity to mammalian cells. Mice immunized with CsuA/B had a survival rate of only 37% when challenged with sublethal doses of *A. baumannii* (43). The survival rate of mice vaccinated with FimA was up to 50% when the mice were challenged with sublethal doses of *A. baumannii* (43). Mice immunized with recombinant of CsuA/B and FimA proteins had a survival rate of 62.5%, which was higher than that of mice immunized with CsuA/B or FimA alone (43). CsuA/B and FimA are candidate subunit vaccines against *A. baumannii* that are highly conserved and have immunogenicity without toxicity. CsuA/B and FimA did not had a strong protective effect on mice, and the probability of clinical translation is low.

#### 2.1.3 Other types of proteins

##### 2.1.3.1 Bap

Biofilm-associated protein (Bap) is found on the surface of bacteria, confers the ability to form biofilms, and plays a relevant role during bacterial infection (111). Bap is a specific bacterial surface protein directly involved in biofilm formation in *A. baumannii* and is involved in intercellular adhesion in mature biofilms (112, 113). Bap is one of the largest bacterial proteins described to date and contains 8621 amino acids. Its predicted isoelectric point (pI) is 2.9, making it one of the most acidic bacterial proteins (112). Bap is composed of seven tandem repeat modules, which are the main components of functional and conserved regions (114). The mice were passively immunized with IgY specifically targeting Bap to prevent *A. baumannii* infection (115). Mice immunized with the recombinant Bap subunit produced high titers of antibodies, which were able to prevent *A. baumannii* infection. The 3-day survival rate of the immunized mice was 60% to 100% (44). Intranasally immunizing mice with chitosan-loaded Bap increased the specific IgG and IgA levels in serum, lung and fecal samples (116). The combined OmpA and Bap vaccines were more potent, with a seven-day survival rate of more than 80% observed in mice when challenged with *A. baumannii* (45). Bap is a candidate subunit vaccine for *A. baumannii*. This protein has high immunogenicity and can protect against *A. baumannii* attack. There is a lack of research on the toxicity of Bap, which is critical for subunit vaccine research.

##### 2.1.3.2 Blp1

The structure of the *A. baumannii* Blp1 protein is similar to that of the giant *A. baumannii* protein Bap. Blp1 is related to the adhesion, virulence and biofilm formation of *A. baumannii* (46, 117). The Blp1 proteins encoded in the genomes of the IC I and IC II strains share 71-74% identity. Purified Blp1 was mildly toxic to lung epithelial cells. Blp1 has a tripartite structure consisting of C-terminal and N-terminal domains with a repetitive region containing multiple motif combinations in the middle. Immunization with the C-terminal fragment of the Blp1 protein protected mice from lethal doses of *A. baumannii*. Immunized mice produced specific IgG antibodies. Mice were injected with a lethal dose of *A. baumannii* intraperitoneally. The seven-day survival rate of actively immunized mice was 60%. Passive immunization by intraperitoneal injection of serum from Blp1-immunized mice provided complete protection to mice (46). Blp1, one of the candidate subunits of *A. baumannii*, is less well conserved than other antigens. Blp1 has some immunogenicity and low toxicity. Blp1-immunized mice showed some protection against *A. baumannii* infection. The probability of clinical translation of Blp1 is not particularly high.

##### 2.1.3.3 VgrG

The type VI secretory system (T6SS) is associated with the virulence and drug resistance of *A. baumannii*. Valine-glycine repeat protein G (VgrG), the core component of the T6SS, is a potent mediator of *A. baumannii* pathogenicity (118, 119). The selected regions of VgrG were conserved in 118 strains of *A. baumannii* (identity ≥97.63%) (47). VgrG is toxic to monocytes and A549 cells. The VgrG1263-1608 sequence was incorporated into the LCL of *N. meningitidis* for surface display and exposure to functional epitopes. The recombinant protein LCL-VgrG was expressed and purified. Mice immunized with LCL-VgrG produced specific IgG antibodies. After challenge with a lethal dose of *A. baumannii*, the survival rates of the mice were 33.3% and 66.6% in the actively and passively immunized groups, respectively (47). Under attack by *A. baumannii*, mice immunized with recombinant VgrG (rVgrG) had higher serum IgG levels, lower bacterial loads in the lungs and spleen, and a 3-day survival rate of 75% (48).

##### 2.1.3.4 MacB

MacAB-tolC is an ABC-type efflux pump responsible for conferring resistance to several antibiotics in bacteria. MacB is an essential control hub in the network and plays a crucial role in the MacAB-tolC efflux pump (120, 121). The MacB protein contains 664 amino acid residues, and 99 homologous sequences were found in *Acinetobacter*, with query coverage and identity greater than 95%. Three epitopes of MacB were screened to form a recombinant epitope (RAE). Mice immunized with RAE produced specific IgG and exhibited increased serum IFN-γ levels. After intraperitoneal injection with *A. baumannii*, the longest survival time of the immunized mice was 14 days, which was significantly higher than that of the control group (23). MacB, a candidate subunit vaccine for *A. baumannii*, is highly conserved and exhibits some immunogenicity. MacB prolonged the survival of infected mice, but did not prevent death, exhibiting little clinical translational value.

##### 2.1.3.5 TolB

TolB is associated with bacterial growth kinetics, motility, and virulence, and is involved in maintaining the integrity of the bacterial envelope (122, 123). TolB is an allosteric β-propeller protein that acts in the bacterial periplasmic space and may interact with other proteins (124–126). Song et al. used bioinformatics software to predict the structure of the TolB protein and indicated that TolB is a potential vaccine antigen. The gene segments of four strains of *A. baumannii* were sequenced, and the similarity of the TolB protein was 96.2%. The T-cell and B-cell epitopes on TolB were screened and reconstituted, and an excellent epitope was designed and verified by experiments (123).

##### 2.1.3.6 CipA and PBP-7/8

CipA and PBP-7/8 are serum drug resistance factors that play a crucial role in the pathogenesis of *A. baumannii* (127, 128). CipA is a plasminogen binding protein. CipA binds to plasminogen and converts it to the active serine protease plasmin, which degrades fibrinogen and complements C3b. CipA directly inhibits the alternative pathway of complement *in vitro* (127). Penicillin-binding protein 7/8 (PGP-7/8) plays a role in cell wall remodeling. Moreover, PBP-7/8 directly or indirectly affects serum drug resistance (128). Seven-day survival rates of mice immunized with CipA, PBP-7/8, and CipA+PBP-7/8 were 60%, 60%, and 80%, respectively, in the *A. baumannii* sepsis model. Immunized mice had higher serum total IgG levels and lower bacterial loads in their spleens than control mice (49). CipA and PBP-7/8 are candidate subunit vaccines against *A. baumannii* that have high immunogenicity and can protect mice against infection. However, its toxicity and conservation remain to be explored.

#### 2.1.4 Novel predicted subunit vaccine candidate proteins

Ahmad et al. used a virome-based reverse vaccinology method to screen two vaccine candidates, polysaccharide export outer membrane protein (EpsA) and chaperone-usher pathway protein B (CsuB). EpsA and CsuB are toxic, antigenic, nonallergenic, and highly conserved (129). Fereshteh et al. showed that Dcap-like proteins and HP2 could be used as vaccine candidates by a reverse vaccinology approach and B-cell epitope analysis. Dcap-like protein and HP-2 have highly conserved surface-exposed epitopes (130). Zadeh Hosseingholi et al. used bioinformatics analysis and found that four hypothetical proteins, HP4, HP6, HP8, and HP15, had the characteristics of vaccine candidates (131). Beiranvand et al. used bioinformatics methods, reverse vaccinology, and subtractive genomics to select five vaccine candidates, namely, Pfsr, LptE, OmpH, CarO and FimF, which have appropriate antigenicity, solubility, and immunogenicity (57, 58).

### 2.2 Polysaccharides

Capsular polysaccharide (CPS) produced by *A. baumannii* surrounds the outer membrane. Composed of repetitive oligosaccharide units (K Units), CPS is a major virulence factor that protects bacteria from environmental damage (132–134). CPS participates in host cell interactions and provides protection against phagocytosis and complement-mediated bactericidal effects (135, 136). CPS can help bacteria evade host immune responses. The pathogen-covering CPS is different from mammalian glycans and therefore unlikely to trigger autoimmunity or allergies in humans (133). Yang et al. passively immunized mice with rabbit polyclonal CPS antibody, which provided 50% protection against *A. baumannii* challenge (136). Three inert carrier proteins were coupled to the type K9 CPS fragment of *A. baumannii*. Immunizing mice with the conjugate induced high levels of IgG antibodies in serum and stimulated the production of IL-10, IL-17A, and TNF-α. Mice immunized with the conjugate together with Freund’s adjuvant and calcium phosphate adjuvant, had 100% and 70% survival after stimulation with *A. baumannii*, respectively (50). Li et al. created a vaccine the glycoprotein CTB4573C‐CPS (C‐CPS) against *A. baumannii* by introducing the O-linked glycosylation system into the host strain. Mice immunized with C-CPS showed a significant increase in the serum-specific IgG antibody level. The survival rate of immunized mice under attack by different *A. baumannii* strains was 70% to 100% (51). CPS has good immunogenicity and can provide protection against *A. baumannii* stimulation, so it is a promising candidate subunit vaccine. Further research is needed on the toxicity of CPS.

### 2.3 Outer membrane vesicles

Outer membrane vesicles (OMVs) are spherical vesicles produced by gram-negative bacteria (137). OMVs play a role in pathogenesis, intercellular communication, and stress responses, as well as in immune regulation and the establishment and balance of the gut microbiota (138). OMVs consist of various proteins (such as OmpA), lipopolysaccharides (LPS), phospholipids, DNA and RNA (139–141). They are naturally occurring candidate subunit vaccines containing multiple candidate vaccine components. Micheal et al. immunized mice with OMVs produced by *A. baumannii* ATCC19606 and produced specific IgG and IgM in serum. In the *A. baumannii* sepsis model, the survival rate of immunized mice was 87.5% to 100%. The bacterial loads in the lungs and proinflammatory factor levels in the serum of immunized mice were reduced compared with those in the control (52). Huang et al. immunized mice with OMVs derived from clinically multi-resistant *A. baumannii* strains. In the sepsis model, the survival rate of actively immunized mice was 73.3% and that of passively immunized mice was 100% (53). Marina et al. immunized mice with LPS-free OMVs. Under challenge with *A. baumannii*, 10 *μg* of OMVs provided 75% protection, and 100 *μg* of OMV provided complete protection (54).

The OMVs produced by *A. baumannii* are toxic. LPS-deficient OMVs have low toxicity, high safety, and high immunogenicity and can protect against *A. baumannii* attacks. LPS-deficient OMVs are promising subunit vaccine candidates with the potential for clinical translation. However, LPS-deficient OMVs are less immunogenic than those produced by wild-type *A. baumannii*. Improving the immunogenicity of LPS-deficient OMVs is a priority. Fusion of immune cell membranes, such as those of neutrophils, with LPS-deficient OMVs may eliminate their toxicity and improve their immunogenicity (142, 143).

## 3 Adjuvants

Adjuvants are a class of substances added to vaccine preparations that improve the immunogenicity of vaccine antigens (144). Reasonable addition of different adjuvants can improve the immune efficacy of vaccines and regulate the immune balance of the body. Adjuvants used in FDA-licensed human vaccines include aluminum salts, MF59, AS01B, AS03, AS04, and CpG ODN (145).

Aluminum adjuvants were the earliest adjuvants to be developed and are the most widely used adjuvants. These adjuvants mainly stimulate humoral immunity, produce a high titer of IgG, and activate Th2 cells (146, 147). Aluminum adjuvants are dispersed in colloidal form in liquids, which is unsuitable for membrane filtration; therefore, membrane filtration cannot guarantee sterility. Aluminum adjuvants also cannot be frozen or lyophilized. Freund’s adjuvant is a water-in-oil emulsion that can cause granulomas and have other adverse effects after injection, so it cannot be used in the manufacture of human vaccines (148). Freund’s adjuvant is still widely used in animal experiments because of its strong adjuvant effect and affordable cost (27, 50, 149). MF59 and AS03 are oil-in-water adjuvants that overcome the shortcomings of Freund’s adjuvant and are effective adjuvants in many vaccines. The fundamental mechanism of action of oil-in-water adjuvant is chemokine-driven cellular immune cell recruitment. MF59 can induce cellular and humoral immunity and produce functional antibodies with high titers. AS03 stimulates the immune system by activating NF-κB, producing proinflammatory cytokines and chemokines, recruiting immune cells (monocytes and macrophages), and inducing high antibody titers (148, 150). CpG ODN is an immune booster that enhances the antibody response and polarizes to the Th1 profile (151, 152). AS01 and AS04 are complex adjuvants, which are combinations of different adjuvants, that enhance the immune response (148). Heat-labile enterotoxin (LT) and cholera toxin (CT) are bacterial toxins extracted from bacteria and are the most promising mucosal adjuvants.

With further in-depth study of adjuvants, especially the development of materials science, the diversity of available adjuvants has greatly increased. Freund’s adjuvant and aluminum adjuvants were the first to be studied, followed composite adjuvants and cytokine adjuvants. Nanomaterials such as CS, PLGA, gold nanoparticles, silver nanoparticles and mesoporous silica have also been shown to have special adjuvant effects (153–158). Bacterial OMVs are natural functional nanomaterials that can be used as drug carriers and adjuvants (159, 160). The OMVs of plants are rarely studied and may also have adjuvant effects. The role of adjuvants has changed from simply inducing innate immunity at the beginning to regulating the level and type of immunity in a systematic manner. Our research team has also explored in *A. baumannii* nanovaccines. The CS-PLGA-rOmp22 nanovaccine was constructed by combining CS, PLGA, and rOmp22 of *A. baumannii*. Immunizing mice with CS-PLGA-rOmp22 greatly enhanced the immune effect (21). Nanomaterials have great application prospects in subunit vaccines and are worthy of further exploration.

## 4 Common immune routes

The common routes of vaccine immunization are intramuscular injection, subcutaneous injection, intraperitoneal injection and mucosal immunization. Muscle tissue is tight, body fluid levels are moderate, and blood vessels are abundant, and these features are conducive to antigen and adjuvant residence. Antigen-induced proinflammatory factors rapidly recruit leukocytes in the blood and lymphatic circulation to infiltrate the injection site (161). Intramuscular injection is easy to perform and widely applicable. Many vaccines are administered intramuscularly, such as the hepatitis B vaccine, and tetanus vaccine. The subcutaneous injection site is located in the connective tissue of the fat layer below the dermis, and the presence of fat can play a significant role in the storage of vaccines (162, 163). Dermal tissue is rich in dendritic cells, macrophages, and a large number of memory T and B cells. Immune cells can take up antigens in subcutaneous tissue through cellular osmosis and present them to the skin for induction of immunity (164, 165). Subcutaneous immunization is suitable for vaccines with a slightly higher dose and a slightly higher risk of adverse reactions. The common vaccines that confer immunity through subcutaneous administration include the inactivated plague vaccine and varicella attenuated live vaccine. The abdominal cavity can accommodate large-volume injections, which are more common for vaccines in animal immunity experiments.

The mucosal immune system is the largest component of the immune system and plays an extremely important role in the process of fighting infection (166). Both mucosal and systemic immune responses can be produced during induction of mucosal immunity. Not only can specific IgA be produced locally but also specific IgG and IgM can be produced in serum (25, 167). A quadrivalent live attenuated influenza vaccine (QLAIV), FluMist/Fluenz, was approved for use in the United States of America (USA) in 2012 and the European Union (EU) in 2013 (168, 169). This representative mucosal vaccine is a nasal spray vaccine. The protective effects of the COVID-19 vaccine and *Pseudomonas aeruginosa* vaccine after nasal mucosal immunization are strong (170–172). Mucosal immunity has the advantages of ease of operation, good safety, relatively few adverse reactions, low cost, and good compliance (173). Mucosal immunity is a promising immunization route, and has been widely studied.

## 5 Animal models

The most relevant experimental animal model should satisfy several key conditions. First, it should offer a heterogeneous immunogenic response. Second, its genetic background should be well defined. BALB/c mice are the most common animals used to evaluate the immunogenicity and protection effects of *A. baumannii* subunit vaccines, C57BL/6, and ICR mice are also used in some research (**Table 1**) (24–26, 30). Some studies have proved that BALB/c and C57BL/6 mice have different immune responses after vaccine administration, Th1 immune response and IFN-γ production dominated in C57BL/6 mice, while BALB/c easily triggered Th2 immune response (174). Floris Fransen et al. indicated that BALB/c, but not C57BL/6 mice had genetic predisposition to produce polyreactive IgAs, has a strong impact on the generation of antigen-specific IgAs (175). We speculate that BALB/c mice are more suitable for *A. baumannii* mucosal immunization vaccine development. It is better to select both BALB/c and C57BL/c mouse models for a more comprehensive assessment of the efficacy of the vaccine.

The specific animal experimental process is shown in **Figure 1**. After immunizing mice subcutaneously, intramuscularly or intranasally with *A. baumannii* subunit vaccine, serum and saliva were collected to detect specific antibodies, immune cells from spleen and lymph nodes were analyzed by flow cytometry, and various cytokines (IFN-γ, IL-4, IL-17) in the spleen supernatant were detected. To test the protective effect of the subunit vaccine, the immunized mice were challenged with *A. baumannii* strains. Common used *A. baumannii* infection models include pneumonia, bloodstream infection, and wound infection models. Clinically, lung infections are more common, such as those contracted during ventilator use, tracheal intubation, and mechanical ventilation. Therefore, the pneumonia model has better application prospects, and it is more meaningful for researchers to use the pneumonia model. After challenged with *A. baumannii* strains, survival rate, weight change, clinical score of the immunized and non-immunized mice, as well as bacterial load and pathology of each organ are observed as protective indicators of *A. baumannii* subunit vaccines.

Bacterial load and pathology injury are important indicators to evaluate the protective efficacy of vaccine. In the pneumonia model, bacterial load in blood and lung tissue and the degree of inflammation in the lung tissue are usually measured. In bloodstream infection model, which is a systemic infection, could affect the main organs, bacterial load in blood, lung, spleen, liver, kidney and heart, and the pathology of lung, spleen and liver are often detected (**Figure 1**). *A. baumannii* bloodstream infection probably causes bacterial endocarditis and produces bacterial valve growth. Although there was no study has examined the pathology of endocarditis, studies indicate that the heart bacterial load in the immunized group was significantly lower than that in the non-immunized group (143, 176). Therefore, selection of indicators based on different infection model is of great significance for the comprehensive evaluation of the protective efficacy of the vaccine.

## 6 Conclusion and prospects

**Figure 3:**
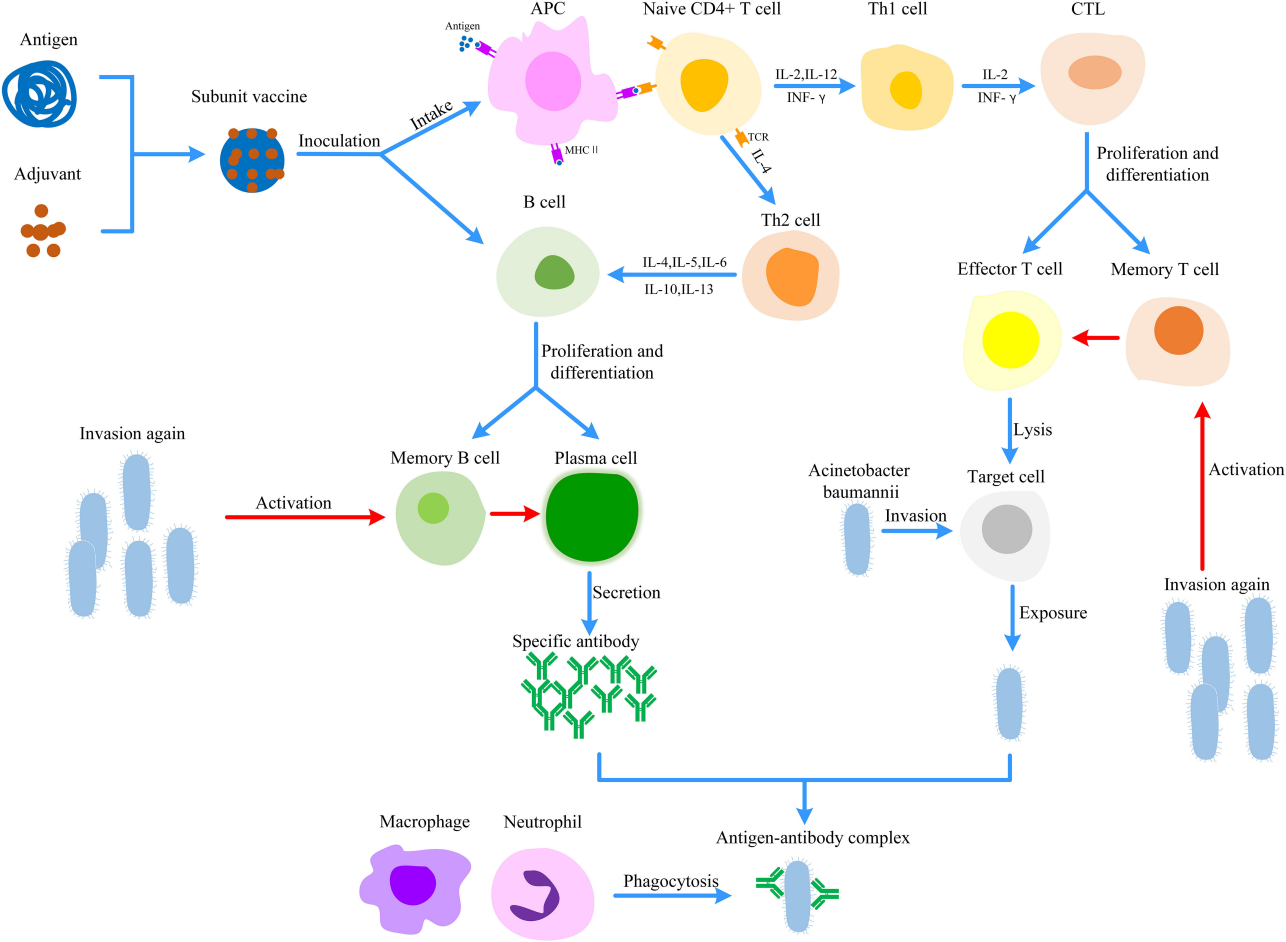
Protective mechanism of *Acinetobacter baumannii* subunit vaccine. Antigens can be taken up and digested into fragments by APC. These antigen fragments are recognized by TCR on CD4+ T cells, which activates cellular immunity to lysis target cells. In parallel, subunit vaccines or cytokines could activate B cells to induce humoral immunity. Once *A. baumannii* invades the body again, memory T cells and B cells could induce rapid and effective immune responses. (APC, Antigen-presenting cell; CTL, Cytotoxic T lymphocyte; MHC, Major histocompatibility complex; TCR, T cell receptor).

*A. baumannii*, as an ESKAPE pathogen, has caused serious harm to global public health (5–8). Vaccines are effective tools to prevent and control *A. baumannii* infection (10). As shown in **Figure 3**, vaccine immunization could induce cellular immunity and humoral immunity, Th1 and Th2 cells secrete IL-4, IL-10, and so on, which activate B cells and induce them to secrete specific antibodies. Meanwhile, a large number of memory T and B cells are generated, which can induce a rapid immune response when exposed to *A. baumannii* again. Subunit vaccines have been widely studied because of their high purity, safety, and stability, ease of production and strong targeted immune response (13). Because of the high cost and time investment associated with the *A. baumannii* subunit vaccine, novel research results have been obtained. Several candidate subunit vaccines have been studied and found to provide partial or complete protection. OmpA, Omp33-36, Ata, LPS-deficient OMVs and BamA are safe, immunogenic and have strong protective effects against *A. baumannii* infection, exhibiting prospects for further development and research.

More and more antigen candidates for the *A. baumannii* subunit vaccine have been predicted and discovered by using proteomics, reverse vaccinology, pan-genomics, core genomics, immunoinformatics, and biophysical analyses (17, 18). Different candidate antigens, such as pure OMPs and detoxified LPS, combined to form a new antigen that can enhance the immune efficacy of vaccines, which is a new idea for vaccine design (19). For particular clinical strains, some unique proteins and metabolic systems involved in the immune escape, nutrient acquisition mechanism, and community interaction of *A. baumannii* may affect the survival of the bacteria. Using these proteins as candidate antigens also offers potential for the development of subunit vaccines (177, 178).

Adjuvants used in FDA-licensed human vaccines include aluminum salts, MF59, AS01B, AS03, AS04, and CpG ODN (145). Freund’s adjuvant is a water-in-oil emulsion that can cause granulomas and other adverse effects after injection, so Freund’s adjuvant cannot be used in the manufacture of human vaccines (148). Freund’s adjuvant is still widely used in animal experiments due to its strong adjuvant effect, immunological enhancement and affordable cost. LT and CT are the most commonly used and promising mucosal adjuvants. Nanomaterials, such as CS, PLGA, gold nanoparticles, silver nanoparticles, mesoporous silicon, and bacterial OMVs, have special adjuvant effects and have attracted the attention of many researchers. The application of nanomaterials as adjuvants in vaccine research is a new and promising direction.

The common methods of vaccine immunization are intramuscular injection, subcutaneous injection, intraperitoneal injection and mucosal immunization. Based on the characteristics of the vaccine, different immunization routes should be chosen. Mucosal immunity is an emerging research hotspot. Mucosal immunity can induce not only the mucosal immune response but also the humoral immune response (25, 167). Mucosal immunity has the advantages of ease of operation, good safety, relatively few adverse reactions, low cost, and good compliance (173).

BALB/c mice, C57BL/6 mice, and ICR mice are three experimental mice of the *A. baumannii* subunit vaccine. Common used animal infection models for *A. baumannii* include pneumonia, bloodstream infection, and wound infection models. Based on the actual situation, different researchers use different animals, infection models and adjuvants. This review summarized the adjuvants, infection models and immunization routes used by different vaccine candidates. By using the same animal, same adjuvant, and same infection model, the immune effects of different vaccines could be compared.

In conclusion, subunit vaccine is one of the effective methods to prevent and control *A. baumannii* infection. So far, no *A. baumannii* subunit vaccine candidate has entered clinical trials. Emerging approaches such as bioinformatics, proteomics, immunoinformatics, biophysical analyses, and reverse vaccinology have played an important role in vaccine candidate screening, protein epitope selection, vaccine spatial structure construction, and vaccine immunogenicity detection. In this review, we summarized the candidate antigens, adjuvants, immunization routes, and animal models for the research of *A. baumannii* subunit vaccines. We also provide opinions and suggestions on novel vaccine development, hoping to guide current and future research on *A. baumannii* subunit vaccines.

## Author contributions

NY primarily wrote the manuscript and drew the figures aided by XD. CZ and FG searched the literatures. XJ and ZW collated the information in **Table 1**, and revised the draft. GF and XD conceived and edited the manuscript. All authors contributed to the article and approved the submitted version.
